# Mass cytometry and transcriptomic profiling reveal body‐wide pathology induced by *Loxl1* deficiency

**DOI:** 10.1111/cpr.13077

**Published:** 2021-06-09

**Authors:** Yu Li, Bingbing Wu, Chengrui An, Deming Jiang, Lin Gong, Yanshan Liu, Yixiao Liu, Jun Li, Hongwei Ouyang, XiaoHui Zou

**Affiliations:** ^1^ Clinical Research Center The First Affiliated Hospital School of Medicine Zhejiang University Hangzhou China; ^2^ Dr. Li Dak Sum & Yip Yio Chin Center for Stem Cell and Regeneration Medicine Zhejiang University Hangzhou China; ^3^ Zhejiang Provincial Key Laboratory of Tissue Engineering and Regenerative Medicine Hangzhou China; ^4^ Zhejiang University‐University of Edinburgh Institute Hangzhou China; ^5^ China Orthopedic Regenerative Medicine Group (CORMed) Hangzhou China

**Keywords:** *Loxl1*, pathology, transcriptome, mass cytometry, hyperplasia, immunity, cancer

## Abstract

**Objective:**

The loss of LOXL1 expression reportedly leads to the prolapse of pelvic organs or to exfoliation syndrome glaucoma. Increasing evidence suggests that *LOXL1* deficiency is associated with the pathogenesis of several other diseases. However, the characterization of the systemic functions of LOXL1 is limited by the lack of relevant investigative technologies.

**Materials and Methods:**

To determine the functions of LOXL1, a novel method for body‐wide organ transcriptome profiling, combined with single‐cell mass cytometry, was developed. A body‐wide organ transcriptomic (BOT) map was created by RNA‐Seq of tissues from 17 organs from both *Loxl1* knockout (KO) and wild‐type mice.

**Results:**

The BOT results indicated the systemic upregulation of genes encoding proteins associated with the immune response and proliferation processes in multiple tissues of KO mice, and histological and immune staining confirmed the hyperplasia and infiltration of local immune cells in the tissues of KO mice. Furthermore, mass cytometry analysis of peripheral blood samples revealed systemic immune changes in KO mice. These findings were well correlated with results obtained from cancer databases. Patients with tumours had higher *Loxl1* mutation frequencies, and patients with *Loxl1*‐mutant tumours showed the upregulation of immune processes and cell proliferation and lower survival rates.

**Conclusion:**

This study provides an effective strategy for the screening of gene functions in multiple organs and also illustrates the important biological roles of LOXL1 in the cells of multiple organs as well as in systemic immunity.

## Graphical abstract

A new method for body‐wide organ transcriptome profiling, combined with single‐cell mass cytometry was developed to perform RNA‐seq of 17 organs from both Loxl1 knockout and wide type (WT) mice reveal body wide pathology (including hyperplasia and infiltration of immune cells) induced by Loxl1 deficiency, which provided a powerful strategy to screen body‐wide organ functions of a particular gene, and also illustrated important biological roles of LOXL1 on multiple organ cells and systemic immunity.

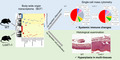

## 1 INTRODUCTION

The extracellular matrix (ECM) constitutes the microenvironment essential for supporting the integrity of tissues and maintaining tissue homeostasis.^1^ Various components of the ECM, such as hyaluronic acid, tenascin‐C, fibronectin and agrin, can differentially regulate cell fate and functions (DNA synthesis, proliferation, migration and differentiation) during regeneration and homeostasis.^2^, ^3^, ^4^ Disruption of ECM components, such as collagen, elastin and hyaluronic acid, can alter the junctional integrity, affect vascular permeability and influence the release of inflammatory factors.^5^ Therefore, these processes are related to diverse pathological conditions and diseases, including embryonic lethality,^6^ inflammatory response and fibrosis,^7^ ageing ^8^ and cancer.^9^

Lysyl oxidase like 1 (LOXL1), an enzyme responsible for elastin synthesis and cross‐linking, is needed for elastic fibre homeostasis and ECM stability. Reportedly, the loss of LOXL1 function leads to pelvic organ prolapse ^10^ and exfoliation syndrome glaucoma.^11^ Increasing evidence suggests that *Loxl1* deficiency contributes to the pathogenesis of several other diseases, such as cancer,^12^ which indicates the importance of LOXL1 and elastin in organ morphology maintenance. However, the characterization of the systemic biological function of LOXL1 and elastin ECM in organs is limited by the unavailability of novel research technologies.

The use of gene knockout (KO) mice for investigating the biological functions of a particular gene primarily focuses on evaluating the visible phenotype in a limited number of organs. *Loxl1* KO mice ^13^, ^14^ have been used to demonstrate that *Loxl1* deficiency leads to abnormal elastin regulation and subsequently leads to pelvic organ prolapse,^15^ flabby skin and bullae.^16^ However, owing to the expression of elastin ECM in various organs, its biological effects and clinical relevance in other organs have always been ignored.

Owing to the significant advances in high‐throughput sequencing technologies, the biological functions of a single gene can now be characterized at the whole‐genome and single‐cell levels.^17^ In this study, we developed an efficient body‐wide organ transcriptomic (BOT) profiling method combined with single‐cell mass cytometry to evaluate the biological manifestations of abnormal elastin regulation through single‐cell mass cytometry and RNA‐Seq of tissues from 17 organs, comparing *Loxl1*(‐/‐) mice with wild‐type mice.

## 2 MATERIALS AND METHODS

### 2.1 Animal model and harvesting of organs

Twelve‐week‐old *Loxl1* KO mice and C57BL/6 mice (both bred at the National Resource Center for Mutant Mice, Model Animal Research Center of Nanjing University) were used in this study. Three animals from each of KO and WT mice groups were humanely killed, and tissue samples were collected from 17 organs (skin, abdominal fat, bone, aorta, brain, lung, bladder, kidney, small intestine, liver, rectum, heart, cartilage, tail tendon, skeleton muscle, vagina and spleen) for the subsequent experiments. Four animals from each group were humanely killed, and the peripheral blood samples were pooled into a single sample for single‐cell mass cytometry.

### 2.2 Ethics Approval

All animal procedures in this study were performed using ethically approved protocols, in accordance with guidelines of The Lab of Animal Experiment Ethical Inspection of College of Medicine, Zhejiang University (Reference number: 2015‐112).

### 2.3 Histological examination

The specimens obtained were immediately fixed in 4% neutral buffered paraformaldehyde, dehydrated by subjecting to an alcohol gradient, cleared and embedded in paraffin blocks. Histological sections (7 μm each) were prepared using a microtome, which were subsequently paraffinized with xylene, hydrated by treating with ethanol at decreasing concentrations and then subjected to haematoxylin and eosin (H&E) staining and Weigart's staining. Next, the sections were mounted and observed under a microscope.

### 2.4 RNA‐Seq

RNA‐Seq was performed according to a previously described method.^18^ Briefly, RNA was extracted from the samples using TRIzol reagent (TAKARA), reverse transcription was performed using SuperScript II Reverse Transcriptase (Invitrogen), double‐strand cDNA was isolated using the NEBNext mRNA second strand synthesis kit, double‐strand DNA was purified using AMPure XP beads (Beckman Coulter), and the sequencing library was constructed using the Nextera XT kit (Illumina), followed by sequencing on the Illumina X‐Ten platform. The RNA‐Seq read data were mapped to the reference genome using TopHat and Cufflinks.^19^, ^20^ The expression levels were calculated and expressed in counts per million.

### 2.5 Data analysis for RNA‐Seq

Differentially expressed (DE) genes were analysed using DESeq2, and data were selected at *P* <.05.^21^ Gene ontology (GO) enrichment analysis was performed using the DAVID informatics resources (https://david.ncifcrf.gov/).^22^

### 2.6 Immunofluorescence staining

The tissues were fixed in 4% (w/v) paraformaldehyde, dehydrated by treating with an ethanol gradient, embedded in paraffin and cut into sections with a thickness of 7 μm. Immunostaining was performed as follows. The paraffinized tissue sections were rehydrated, and the antigens were retrieved, followed by rinsing three times with PBS. Next, the sections were treated with blocking solution (1% BSA) for 30 min before overnight treatment with primary antibodies at 4℃. Rabbit anti‐mouse antibodies against KI67 (Abcam, ab16667) and CD45 (BD Biosciences, 555 483) and rat anti‐mouse antibodies against CD19 (Biolegend, 115 525) and F4/80 (Biolegend, 123 121) were used as primary antibodies to detect cell proliferation and immune cell infiltration. The secondary antibodies goat anti‐rabbit Alexa Fluor 488 (Invitrogen, A11008), goat anti‐rabbit Alexa Fluor 546 (Invitrogen, A21430‐f) and goat anti‐rat CY3 (Beyotime Institute of Biotechnology, A0507) and DAPI (Beyotime Institute of Biotechnology, China) were used to visualize the respective primary antibodies and cell nuclei. All procedures were performed according to the manufacturer's instructions.

### 2.7 Single‐cell mass cytometry

Blood samples from four mice from each group were pooled to obtain sufficient cells for reliable mass cytometry. After lysing the erythrocytes using ACK lysis buffer, the samples were washed with FACS buffer and incubated at 4℃ or on ice. The pooled cells were then stained with cisplatin to distinguish between live and dead cells. After blocking at room temperature for 20 min, the cells were stained with a mixture of metal‐tagged antibodies targeting surface antigens for 30 min at room temperature (the complete list of antibodies is provided in Table S1). After washing with FACS buffer and fixation for 20 min at room temperature, the cells were washed with Perm buffer and stained using a mixture of intracellular antibodies (Table S1) for 30 min at room temperature. Next, the cells were fixed and stained with a DNA intercalator overnight at 4℃. After washing with Perm buffer, the cells were incubated with barcodes for 30 min at 4℃. After multiple washes with FACS buffer and ultrapure H_2_O, the cells were analysed using a CyTOF mass cytometer. The raw data acquired were uploaded to a Cytobank web server (Cytobank Inc) for further data processing and for gating dead cells and normalization beads.

### 2.8 Mass cytometry data analysis

Data analysis was performed using the SPADE ^23^ algorithms and a scatter diagram in Cytobank (www.cytobank.org). SPADE analyses were performed in Cytobank with the target number of nodes set to 200. In SPADE algorithms, the colour gradient represents the median expression level of the selected marker. The following markers were selected for immune response‐related clustering: CD45, CD3, CD4, CD8, gdTCR, IgM, CD11b, Ly6G, Ly6C, F4/80 and CD49b. Cell populations were identified based on the marker expression patterns shown in the figure. The percentage of each immune cell population was calculated for each defined population in the total CD45+ cell population. The intensities of the markers were calculated using transformed median intensity values in each defined cell population. Heat maps were constructed using the heat map illustrator HemI 1.0.3.3.^24^

### 2.9 Statistical analysis

A two‐tailed t test was used to detect differences in histological results between the KO and WT mice groups. Statistical significance was set at *P* <.05.

## 3 RESULTS

### 3.1 Body‐wide pathology induced by *Loxl1* deficiency

**FIGURE 1:**
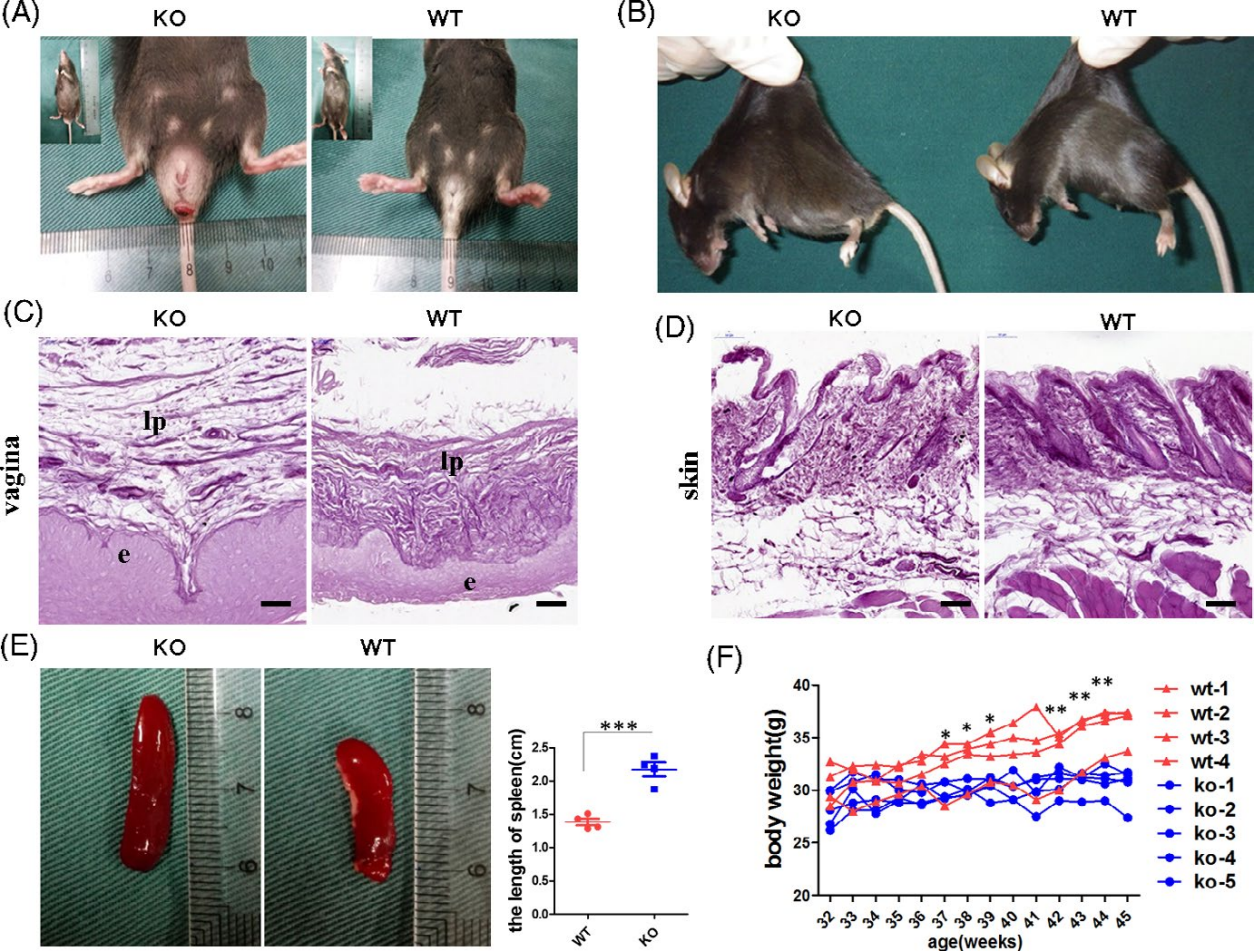
Abnormal elastin‐associated phenotype and other systemic changes in *Loxl1* knockout (KO) mice. A, Overview of the pelvic floor phenotype in *Loxl1* KO (left) and wild‐type mice (right). B, Overview of loose skin in KO mice. C, D, Weigart's staining for elastic fibre visualization in the vagina (C) and skin (D), scale bar, 50 μm. E, Left: overview of the spleen. Right: measurement of the length of the spleen; the data represent mean ±s.d. for four samples. ***P <.001. F, The body weights of *Loxl1* KO and WT mice; the line represents data of each mouse. **P <.01, *P <.05

As LOXL1 is the key enzyme for the synthesis and assembly of elastin, *Loxl1* KO mice exhibited phenotypes associated with abnormal elastic fibres, including obvious POP (Figure 1A) and loose skin (Figure 1B). POP is characterized by an enlarged perineal body and a bulge in the rectum and vagina. In humans, the molecular mechanism underlying POP is widely considered to be related to the theory of ‘imbalance of elastin’ ^25^; therefore, *Loxl1* KO mice are always used as an animal model for studies on POP. To observe changes in elastic fibres in KO mice, we performed Weigart's staining specific for elastic fibres in vaginal tissues (Figure 1C) and skin (Figure 1D). In the KO mice, elastic fibres in the lamina propria of the vagina and in the skin appeared to have a thin and short rod‐shaped structure, and the deposition of elastic fibres near the basement membrane decreased in the KO mice model. In contrast, the elastic fibres were polarized and arranged neatly in the vaginal tissues of WT mice, confirming the reduction of synthesis and failure of cross‐linking process of elastic fibres in KO mice. Moreover, the spleens of KO mice were longer and had a larger volume than those of WT mice (Figure 1E). The body weight of KO mice decreased significantly compared with that of WT mice with increasing age (Figure 1F). The observation of these additional phenotypic changes suggested that the effects of *Loxl1* deficiency were not limited to the reproductive tracts and also affected the spleen and other organs in mice.

### 3.2 Upregulation of inflammation and hyperplasia in *Loxl1*‐deficient mice revealed using body‐wide organ RNA‐Seq

**FIGURE 2:**
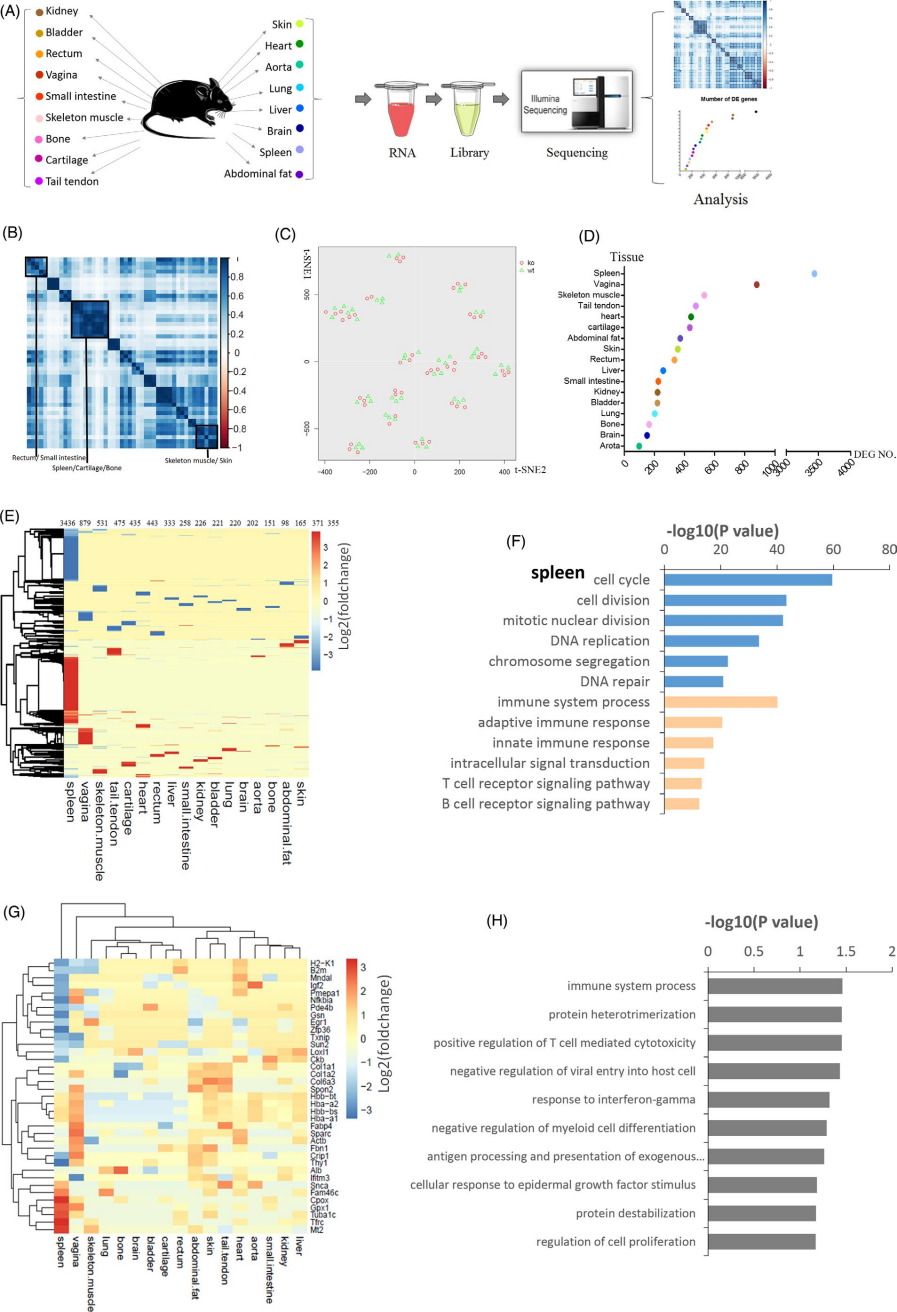
Detection of upregulated inflammation and proliferation at the transcriptome level through body‐wide organ RNA‐Seq. A, Scheme illustrating body‐wide organ RNA‐Seq; there were three animals in the knockout (KO) group as well as in the wild‐type group, and 17 organs were harvested from each animal. B, Heat map of the pairwise correlation among the transcript levels of 46,814 genes in the 17 tissue types from the WT group. C, tSNE clustering analysis of the body‐wide organ mRNA profiles for the KO and WT groups. D, Scatter plot of the number of significantly differentially expressed (DE) genes in the 17 tissue types. E, Heat map of the DE genes in all tissues ordered by hierarchical clustering. The log2 (fold change) values relative to the corresponding values in the WT group are shown (*P* <.05). F, Biological process gene ontology (GO) analysis of DE genes in the spleen using DAVID software. The GO terms in blue and orange represent gene sets upregulated and downregulated, respectively, in the KO group compared with that in the WT group. The six most significant gene sets per condition are shown. G, Heat map of the common DE genes (shared in ≥five tissues) ordered by hierarchical clustering. The log2 (fold change) values relative to the values in the WT group are shown (*P* <.05). H, Biological process GO analysis of common DE genes using DAVID software

To assess the systemic effects of abnormal elastin production on organs and tissues in *Loxl1* KO mice, we performed RNA‐Seq using tissues collected from 17 organs (heart, liver, spleen, lung, kidney, skin, brain, aorta, abdominal fat, small intestine, bladder, rectum, vagina, skeletal muscle, bone, cartilage and tail tendon) harvested from WT and KO mice, with biological triplicates for each group (Figure 2A). We first performed correlation analysis of gene expression for all samples from the WT group. The data revealed a high correlation between the expression profiles of similar tissue types. Tissues from organs that are closely associated, such as the rectum and small intestine, cartilage and bone, and skeletal muscle and skin, could be clustered based on gene expression, indicating good repeatability in the sequencing results (Figure 2B). Principle component analysis was performed. Figure 2C shows the tSNE results of this data, which indicate a clear distinction in the gene expression profiles between the KO and WT mice groups. This indicates the differential characteristics of the two groups (Figure 2C).

To further compare the DE genes in various tissues in the two groups, we used DESeq2 in R (http://www.R‐project.org) for performing downstream analyses (Figure 2D). The data indicated that the spleen had the highest number of significantly differentially expressed genes (*P* <.05), followed by the vagina and skeletal muscle, suggesting that *Loxl1* deficiency affects the spleen most significantly. As indicated in the heat map generated for DE genes in all tissues (Figure 2E), most DE genes were unique to each tissue type, which implied that *Loxl1* deficiency exerts tissue‐specific effects. GO analysis (https://david.ncifcrf.gov/) of the DE genes in different tissues also confirmed the tissue‐specific changes following *Loxl1* KO. For example, DE genes upregulated in the spleen were enriched with genes associated with the cell cycle and cell division after *Loxl1* KO, whereas the downregulated DE genes were those associated with the immune response (Figure 2F). In contrast, genes encoding proteins related to keratinization and keratinocyte differentiation were upregulated, whereas those encoding proteins related to sarcomere organization and response to estradiol were downregulated in the vagina (Figure S1). In skeletal muscles, the upregulated GO terms included mitotic nuclear division and mitotic cell cycle, and the downregulated GO terms included immune system process and antigen processing and presentation (Figure S1).

In addition to the tissue‐specific changes in different tissue types, we identified 38 common DE genes in more than five tissues (Figure 2G); GO enrichment with cell component analysis indicated that the common upregulated genes were primarily associated with extracellular exosomes (20/38) (eg, *Col1a1*, *Col6a3*, *Col1a2*, *H2k1*, *Actb*, *Ifitm3* and *Fbn1*), cytoplasmic components (23/38) (eg, *Zfp36*, *Txnip*, *Egr1*, *Actb*, *Crip1*, *Ifitm3*, *Mndal*, *Snca* and *Nfkia*), nuclear components (17/38) (eg, *Zfp36*, *Txnip*, *Egr1*, *Ifitm3*, *Snca*, *Mndal* and *Sun2*) and basement membrane (4/38) (*Alb*, *Fbn1*, *Sparc* and *Loxl1*). Additionally, the GO terms of biological process analysis also indicated that these DE genes were enriched in ‘immune system process’, ‘response to bacterium’ and ‘cell proliferation’ (Figure 2H), which indicated that multiple tissues underwent common significant changes during the immune process and cell proliferation at the transcriptome level.

### 3.3 Tissue hyperplasia and local immune cell infiltration in KO mice

**FIGURE 3:**
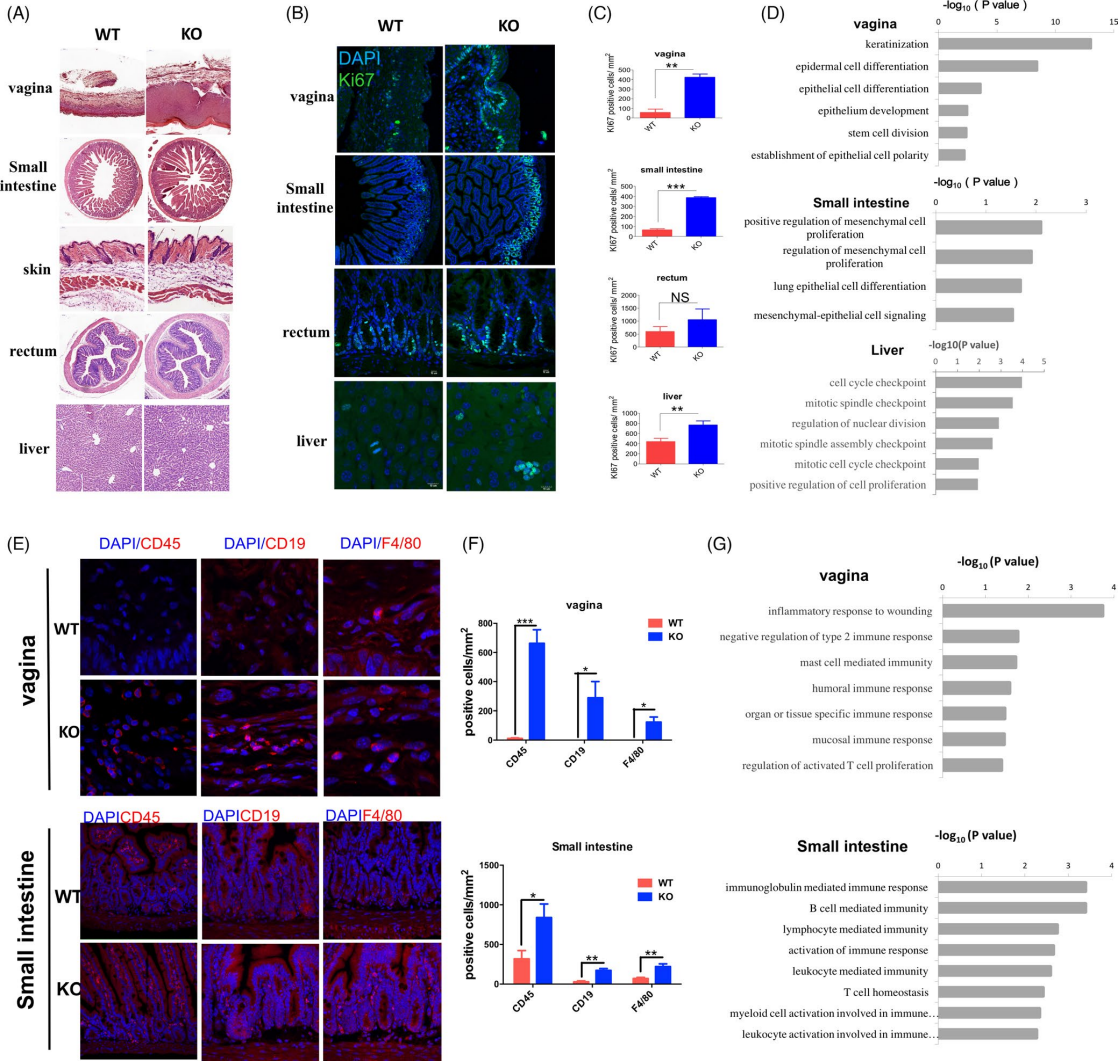
Tissue hyperplasia and local immune cell infiltration in knockout (KO) mice. A, H&E staining of multiple tissues in wild‐type and KO mice, including the vagina, small intestine, skin, rectum and liver. B, immunofluorescence staining for KI67 in the tissues of the vagina, small intestine, rectum and liver in WT and KO mice. C, Bar graphs showing the number of KI67+ cells per square millimetre in tissues of the intestine, skin, rectum and liver. The data represent mean ±s.d. of three samples. ***P <.001, **P <.01, NS, no significance. D, Gene ontology (GO) terms of differentially expressed (DE) genes associated with cell proliferation in multiple tissues, including those from the vagina, small intestine and liver, in WT and KO mice. E, Immunofluorescence staining of immune cell markers (CD45 for immune cells, CD19 for B cells and F4/80 for macrophages) in vaginal and small‐intestinal tissues in WT and KO mice. F, Bar graphs showing the number of CD45+, CD19+ and F4/80+ cells per square millimetre in vaginal and small‐intestinal tissues. The data represent mean ±s.d. of three samples. ***P <.001, **P <.01, *P <.05. G, Gene ontology (GO) terms of DE genes associated with immune responses in the vagina and small intestine

To confirm the changes in cell proliferation in KO mice, histological staining was performed to further analyse all tissue samples. H&E staining showed that the vaginal epithelium was thicker and the villi of the small intestine were longer in KO mice than in WT mice (Figure 3A); in contrast, other tissues, including those from the skin, rectum and liver, showed no apparent changes.

Next, we performed immunofluorescence staining using the proliferation marker KI67. Compared with the WT mice, the KO mice had higher levels of KI67 expression in the vaginal basal lamina, liver and crypts of the small intestine (Figure 3B, 3C). This was consistent with the transcriptomic data, in which GO terms related to cell proliferation (Figure 3D), including keratinization, epidermal cell differentiation and positive regulation of mesenchymal cell proliferation, were upregulated. Immunofluorescence staining for KI67 was also repeated using the remaining tissues; however, no differences were observed between the two groups.

Subsequently, immunofluorescence staining was performed using anti‐CD45, anti‐CD19 and anti‐F4/80 antibodies to detect the local infiltration of immune cells. In the KO mice group, the number of CD45+, CD19+ and F4/80+ cells increased in the vagina and small intestine (Figure 3E, 3F); this was consistent with the transcriptomic data, in which GO terms related to immune processes were upregulated (Figure 3G), indicating the local infiltration of immune cells (leukocytes, B cells and macrophages). These results suggest that *Loxl1* deficiency induces hyperplasia and local immune cell infiltration in specific tissues.

### 3.4 Systemic immune changes in KO mice detected using CyTOF (mass cytometry)

**FIGURE 4:**
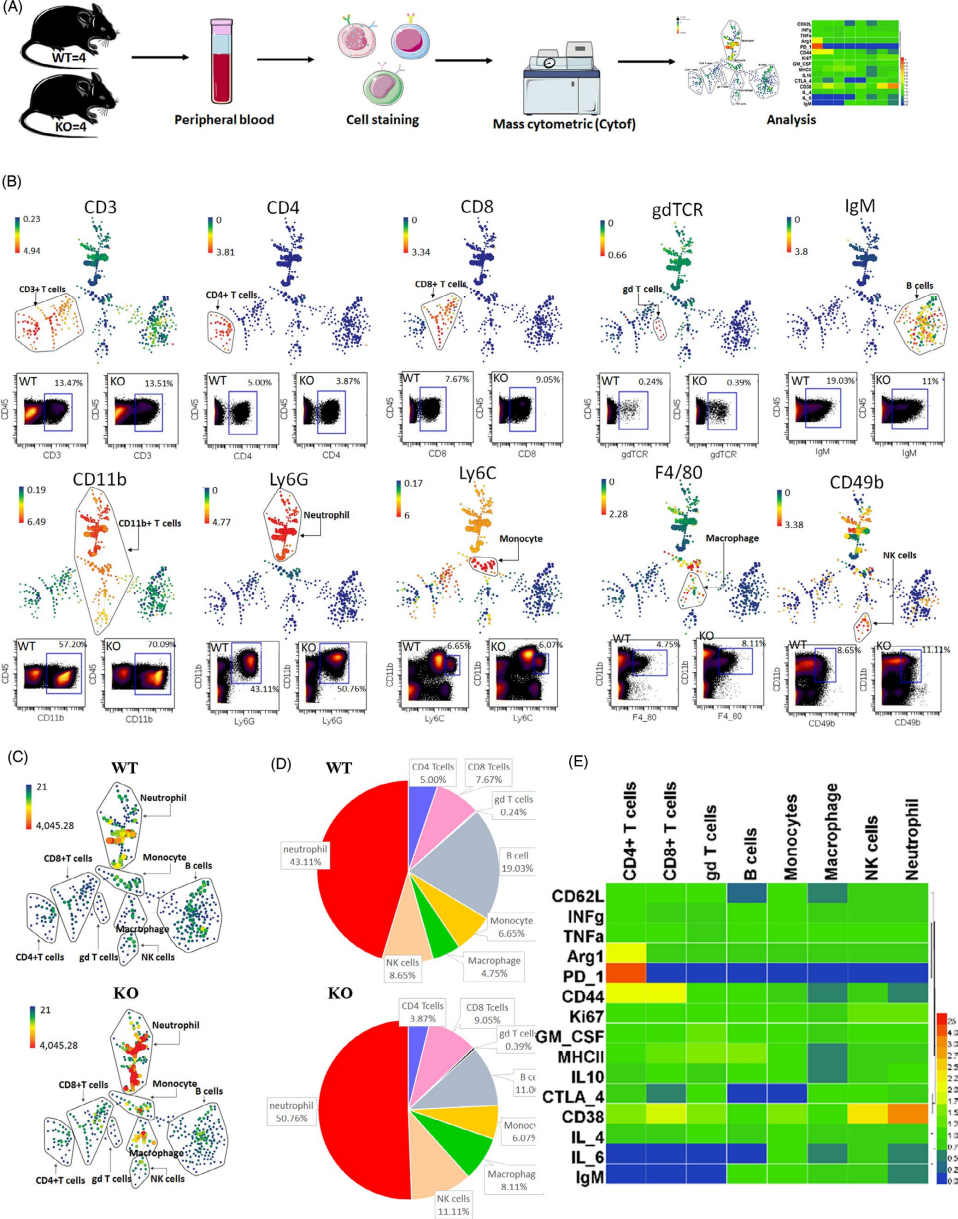
Systemic immune changes in knockout (KO) mice. A, Scheme of the experimental procedure for the characterization of immune cell populations in peripheral blood samples using mass cytometry. Peripheral blood extracted from four mice from each group was pooled into a single sample and stained using a cocktail of metal‐tagged antibodies. The samples were analysed using a CyTOF instrument, and the immune cell populations were identified using SPADE and standard dot plots. B, SPADE analysis for the characterization of immune cell populations using canonical markers, including CD3, CD4, CD8, gdTCR, IgM, CD11b, Ly6G, Ly6C, F4/80 and CD49b. In SPADE, the node size correlates with the number of cells, and the coloured gradient corresponds to the arcsinh‐transformed expression of the median expression value. Dot plots representing marker labelling on CD45+ cells and the proportion of each cell population in the wild‐type and KO groups are shown. C, SPADE diagram of the identified immune cell populations in the WT and KO groups. D, The relative abundances of the identified immune cell populations in the WT and KO groups. E, Heat map of immune cell functional marker expression. The values represent fold changes in immune cell marker expression in the KO group compared with that in the WT group

Based on the upregulation of immune‐related GO terms detected using RNA‐Seq, we predicted changes in the systemic immunity of KO mice. To evaluate this, we performed CyTOF for characterizing the immune cell populations in peripheral blood samples from WT and KO mice using 32 cell markers. This was performed to assess the effect of *Loxl1* inefficiency on systemic immunity. The antibody panel targeted major innate and adaptive immune cell subset markers, including cell surface markers, functional markers and cytokines. CyTOF data were processed using Cytobank (www.cytobank.org) and SPADE, which are tools used for the visualization of complex cell composition and proportion in the peripheral blood (Figure 4A).

Well‐recognized cell surface markers were used to identify specific cell subsets in the peripheral blood (Figure 4B). We first performed gating to obtain CD45+ cells from the peripheral blood samples (Figure S2A); SPADE analysis revealed that the gated cells were all CD45+(Figure S2B). We then identified major cell subpopulations within the CD45+ cell population (Figure 4B, 4C); among the CD3+ cells, those that expressed CD4, CD8 and gdTCR were characterized as CD4+ T cells (CD3+CD4+), CD8+T cells (CD3+CD8+) and gd T cells (CD3+gdTCR+), respectively. We also identified B cells (CD45+IgM+) and four subsets of cells expressing CD11b at high levels, including neutrophils (CD45+CD11b+Ly6G+), monocytes (CD45+CD11b+Ly6C+), macrophages (CD45+CD11b+F4/80+) and NK cells (CD45+CD11b+CD49b+).

The proportion of each immune cell subset with respect to the entire CD45+ cell population in the WT and KO groups was then compared with assess the effect of *Loxl1* KO on the immune system (Figure 4D). The WT and KO groups had the same proportion of CD3+ T cells (WT, 13.47%; KO, 13.51) and monocytes (WT, 6.65%; KO, 6.07%), whereas the ratio of CD4+ T cells (WT, 5.00%; KO, 3.87%) and B cells (WT, 19.03%; KO, 11%) was marginally lower in the KO group. Moreover, the proportion of CD8+ T cells (WT, 7.67%; KO, 9.05%), gd T cells (WT, 0.24%; KO, 0.39%), neutrophils (WT, 43.11%; KO, 50.76%), macrophages (WT, 4.75%; KO, 8.11%) and NK cells (WT, 8.65%; KO, 11.11%) in peripheral blood samples was relatively higher in KO mice than in WT mice. Therefore, *Loxl1* deficiency influenced the immune system in mice, as indicated by alterations in the proportion of immune cell subsets, including the increased ratios of neutrophils, NK cells, macrophages and CD8+ T cells and decreased ratios of B cells and CD4+ T cells.

The applied antibody panels also included various other functional markers, including those for antigen presentation and costimulation (major histocompatibility complex, MHC‐II), cell activation and cell phenotype (CD44, IgM and Arg1), cell migration (CD62L), cell signalling transduction (CD38), cytokines (IL‐4, IL‐6, IL‐10 and GM‐CSF), tumour necrosis factor alpha (TNF‐α), interferon gamma (IFN‐γ) and immunosuppressive factors (CTLA‐4 and PD‐1). The heat map, a visualization tool, was used to display the fold changes in the levels of functional markers in different cell subsets in KO mice relative to those in WT mice (Figure 4E). The heat map showed that while CD4+ T cells showed higher expression of Arg1, PD‐1 and CD44, CD8+ T cells showed higher expression of CD44 and CD38, and the NK cells showed higher expression of CD38 (fold change >2). Meanwhile, the fold changes in the levels of CTLA4 in CD8+ T cells, CD62L in B cells, CD62L, CD44, MHCII, IL10 and IL6 in macrophages, and CD44 and IL6 in neutrophils were lower (fold change <0.5). However, the difference in the expression levels between the two groups was negligible for most genes. Nevertheless, these results indicated that T cells and NK cells were activated in *Loxl1* KO mice, whereas B cells, macrophages and neutrophils exhibited lower levels of migration and secretion in the KO mice group.

### 3.5 Clinical relevance between *LOXL1* mutation and cancer

**FIGURE 5:**
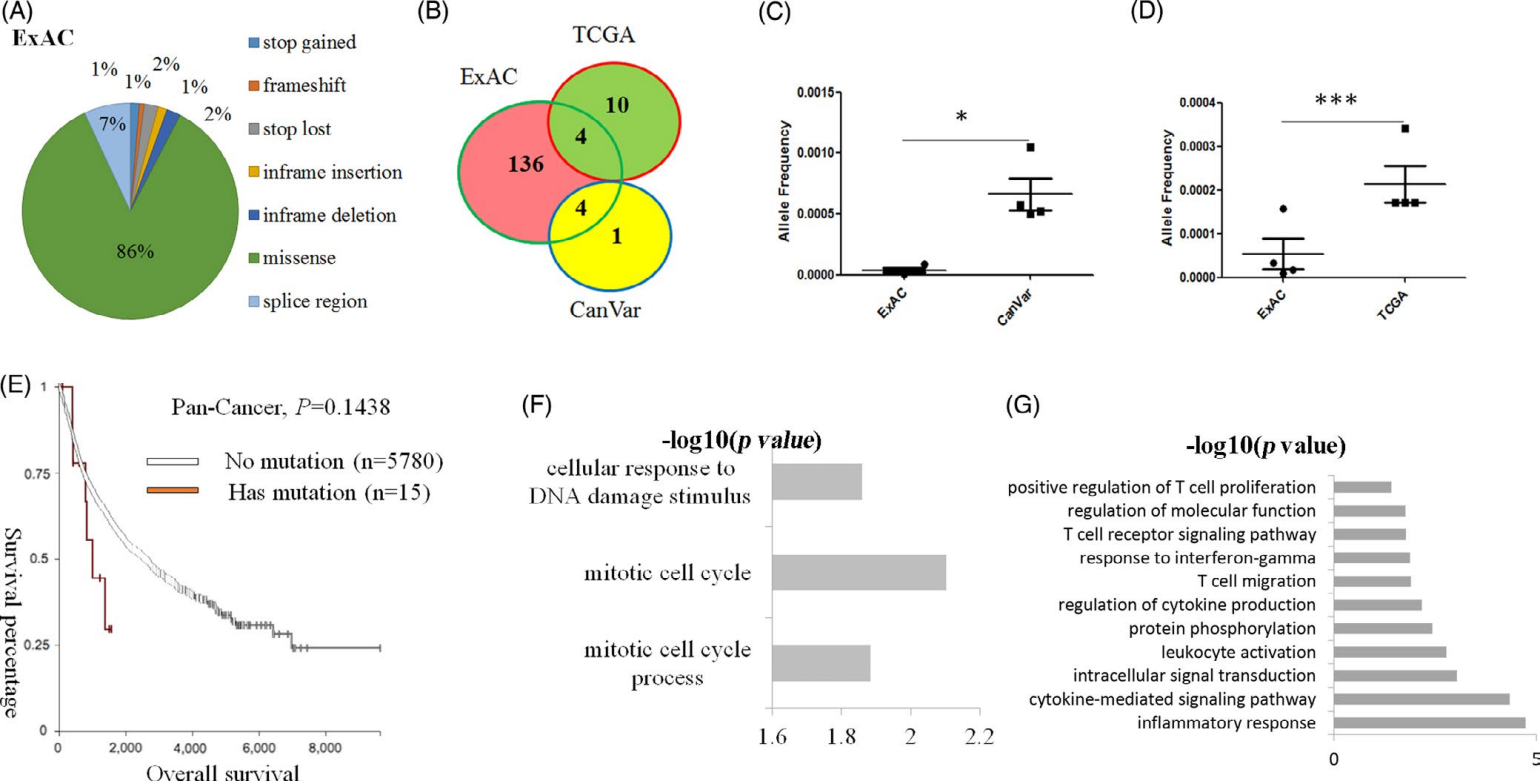
Clinical data for the relationship between *LOXL1* mutation and cancer. A, Variations in *LOXL1* in a specific population. B, Common mutation loci of *LOXL1* in the general population from ExAC compared with that in patients with cancer from TCGA and CanVar. C, D, Mutation frequency in the common mutation loci of *LOXL1* in the general population from ExAC compared with that in patients with cancer from CanVar (C) and TCGA (D), respectively. E, Overall survival rate of patients with *LOXL1*‐mutant tumours (n = 15) compared with that of patients with *LOXL1*‐non‐mutant tumours (n = 5780) in the pan‐cancer patient population. F, Biological process gene ontology (GO) analysis of upregulated genes in patients with *LOXL1*‐mutant hepatocellular carcinoma using DAVID software. G, Biological process GO analysis of upregulated genes in patients with *LOXL1*‐mutant lung adenocarcinoma using DAVID software

As *LOXL1* deficiency can induce changes in the immune system and tissue hyperproliferation, which are the hallmarks of tumours,^26^ we hypothesized that mutations in *LOXL1* are correlated with cancer progression. To confirm this, we first obtained data from the Exome Aggregation Consortium (ExAC, http://exac.broadinstitute.org/gene/ENSG00000129038) to identify *LOXL1* mutations in a common human population. According to information from the database, the studied population had multiple sequence variations (including missense mutations, frameshift mutations, large fragment deletions, large fragment insertions and early termination, among others) in the *LOXL1* gene. Missense mutations accounted for approximately 86% of the variations (Figure 5A) and led to disruption of polypeptide chain functions.

As the data set provided on ExAC includes data for 60,706,000 unrelated individuals and can serve as a useful reference set of allele frequencies for studies on severe diseases, we next used the data set from ExAC as a control group representing the general population and used gene mutation datasets from TCGA and CanVar (https://canvar.icr.ac.uk/gene/ENSG00000129038), which are two ontology databases, as tumour patient groups. There were four common loci of *LOXL1* mutations in ExAC in the general population compared with that in patients included in TCGA and CanVar (Figure 5B). Additionally, the mutation frequencies of the eight loci were significantly higher in patients with tumours than in the general population (Figure 5C, 5D), which indicated that a higher *LOXL1* mutation frequency may be associated with tumorigenesis.

Next, we compared the overall survival rates between patients with *LOXL1*‐non‐mutant and *LOXL1‐*mutant tumours from TCGA. Among the pan‐cancer patients, the overall survival rate in patients with *LOXL1‐*mutant tumours (n = 15) was relatively lower than that in patients with non‐mutant tumours (n = 5780) (*P* =.1438) (Figure 5E).

The GO analysis revealed that among patients with hepatocellular carcinoma, the cell cycle‐related GO terms were significantly upregulated in patients with *LOXL1*‐mutant tumours than in patients with *LOXL1*‐non‐mutant tumours, and similar outcomes were observed in GO terms related to mitotic cell cycle, mitotic cell cycle process and cellular response to DNA damage (Figure 5F). In patients with lung adenocarcinoma, GO terms related to immune activation, including T‐cell proliferation, T‐cell migration, signal transduction and cytokine production, were significantly upregulated in patients with *LOXL1*‐mutant tumours (Figure 5G). Therefore, compared to patients with *LOXL1*‐non‐mutant tumours, patients with *LOXL1*‐mutant tumours not only had a lower survival rate but also higher levels of transcripts of genes associated with biological processes, such as cell proliferation and immune activation. These results are consistent with the phenotypes identified in *Loxl1* KO mice.

## 4 DISCUSSION

Our findings showed that abnormal elastin activity induced by *Loxl1* deficiency can induce a series of tissue‐specific elastin‐related modifications. Using high‐throughput sequencing combined with single‐cell CyTOF and histological evaluation, we demonstrated that *Loxl1* deficiency in mice promoted local immune responses and hyperplasia in multiple tissues and also induced drastic changes in systemic immunity functions. Lastly, we demonstrated the correlation among *Loxl1* deficiency, tumorigenesis and poor survival rate in patients with tumours.

ECM remodelling contributes to the progression and development of diseases.^4^ The RNA‐Seq data revealed that most significantly upregulated DE genes (20/38) encoded ECM components (*Fbn1*, *Col6a3*, *Co1a2*, *Col1a1* and *Loxl1*), which indicates that *Loxl1* deficiency can induce elastin fragmentation and subsequent ECM remodelling. The results from our model also suggested that elastin fragmentation and ECM remodelling led to the activation of major chemotactic activities, recruitment of monocytes and macrophages to the site of local inflammation in multiple organs and initiation of a systemic immune response in the peripheral blood. Several reports have shown that aberrant fragmentation and ECM component expression can affect cell activation and survival, leading to inflammation and immune response activation.^27^ The matrix fragment of collagen (the tripeptide N‐acetyl Pro‐Gly‐Pro) plays an important role neutrophil chemoattraction owing to its ability to mimic the chemotactic properties of CXCL8.^28^ Other constituents of the ECM, such as hyaluronan, induced the perpetuation of inflammatory responses through the activation of TLR2, TLR4 or both.^29^ Fibulin‐5 can affect cutaneous inflammation by regulating the NF‐κB levels in active inflammation.^30^ Therefore, in *Loxl1* KO mice, the promotion of the local infiltration of monocytes and macrophages may have been mediated through chemokine‐ and cytokine‐induced immunity.

The immune system plays a crucial role in tissue homeostasis. For example, several studies have shown that the activation of inflammatory responses is closely associated with tissue hyperplasia. Zhong et al showed that IL1 secreted by activated monocytes/macrophages can promote epithelial cell proliferation and increase epithelial layer thickness through paracrine action.^31^ Furthermore, in colitis, inflammatory mediators promote cell proliferation through the PI3K/Akt/β‐catenin pathway.^32^, ^33^ Members of the NF‐κB ^34^ family and their inhibitors can help maintain cell viability and proliferation through the IKK/IκB/NF‐κB pathway.^35^ Overall, these results indicate that hyperplasia occurring in multiple tissues in the *Loxl1* KO mice model may have been mediated and followed by local immune infiltration and induction of systemic immunity.

Our results also revealed a close relationship between *Loxl1* mutation and tumour susceptibility, with a higher mutation frequency and lower overall survival rate observed in patients with *Loxl1* mutations. The specific cell cycle phase and inflammation in the cellular microenvironment are closely related to cancer progression and outcome ^36^ as an inflamed microenvironment can disrupt the quiescence of tumour stem cells and initiate tumorigenesis,^37^, ^38^ which is consistent with our finding that *Loxl1* KO induces local and systemic immune responses as well as multiple tissue hyperplasia. Previous studies have also shown that increased proliferation and self‐renewal play a key role in tumorigenesis. The high rate of DNA replication during proliferation makes the process more error‐prone. Among all cancer‐related mutations, 66.1% were attributed to random DNA replication errors (39). Increased mutation rates and genomic instability are inherent characteristics of tumour progression, and an increase in *LOXL1* mutations may simply be a red herring rather than a cause of cancer progression. Therefore, further studies are required to confirm whether *Loxl1* KO mice have a higher random mutation rate.

## 5 CONCLUSION

We developed a strategy by combining single‐cell mass cytometry and BOT analysis to evaluate the effect of *Loxl1* KO on systemic immunity and tissue hyperplasia in mice. Our findings provide a powerful strategy for screening the functions of a particular gene in multiple organs as well as the important biological roles of elastin in various cell types and in systemic immunity. These findings may be considered crucial for understanding ECM biology and pathology.

## CONFLICT OF INTEREST

All authors have agreed to the publication of this paper and declare no potential conflicts of interest.

## AUTHOR CONTRIBUTIONS

XHZ had full access to all the data in the study and takes responsibility for the integrity of the data and the accuracy of the data analysis. Study concept and design: HO, YL and BBW. Acquisition of data: DMJ, LG, YSL and YXL. Analysis and interpretation of data: CRA and BBW. Drafting of the manuscript: YL, BBW, and JL. Critical revision of the manuscript for important intellectual content: XHZ and HO.

## Supporting information

Figure S1

Figure S2

Table S1

## Data Availability

All data in this paper are deposited in a public database and will be made available upon publication.
