# Supplementary figures and images for: Mass cytometry and transcriptomic profiling reveal body‐wide pathology induced by Loxl1 deficiency
Source: Cell Prolif. 2021 Jun 9;54(7):e13077. doi: 10.1111/cpr.13077 (PMC8249785; doi:10.1111/cpr.13077)

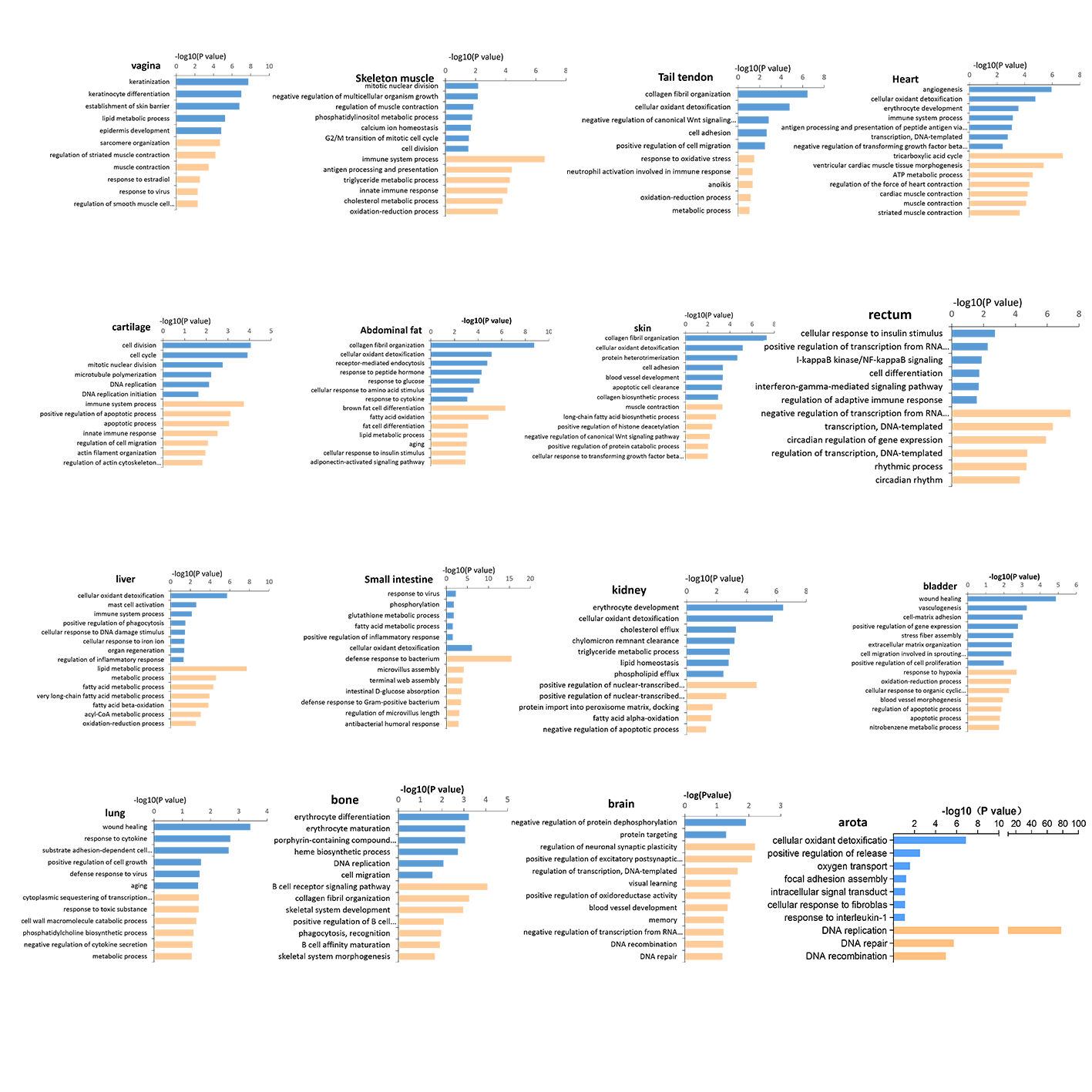

Supplement: Supplementary file 1 — Figure S1 [file CPR-54-e13077-s003.tif]

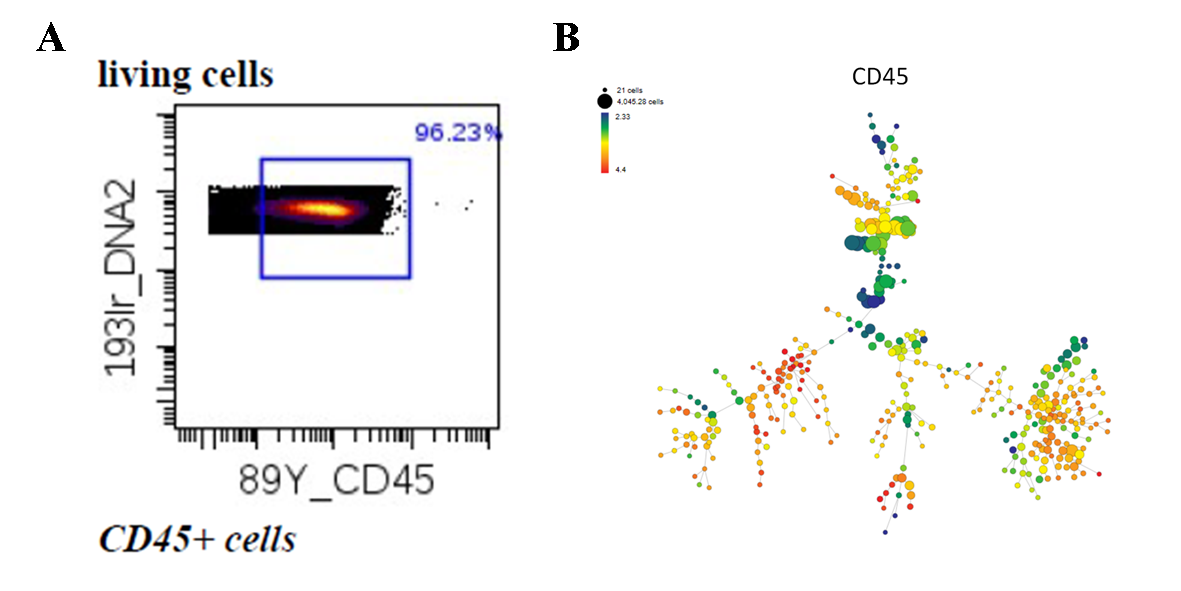

Supplement: Supplementary file 2 — Figure S2 [file CPR-54-e13077-s002.tif]
