# Supplementary material for: Mass cytometry and transcriptomic profiling reveal body‐wide pathology induced by Loxl1 deficiency
Source: Cell Prolif. 2021 Jun 9;54(7):e13077. doi: 10.1111/cpr.13077 (PMC8249785; doi:10.1111/cpr.13077)
Supplement: Supplementary file 3 — Table S1 [file CPR-54-e13077-s001.docx]

**Supplement table1: Antibodies used in CyTOF**

| **List** | **Channel** | **Antibody** | **Clone** | **Brand** | **Cat #** |
| --- | --- | --- | --- | --- | --- |
| 1 | 158Gd | CD3 | 145-2C11 | Biolegend | 100302 |
| 2 | 197Gd | CD4 | RM4-5 | Biolegend | 100520 |
| 3 | 115In | CD19 | 6D5 | Biolegend | 115502 |
| 4 | 153Eu | gdTCR | GL3 | Homemade | / |
| 5 | 198Pt | CD8 | 53-6.7 | Biolegend | 100716 |
| 6 | 113In | CD44 | IM7 | Biolegend | 103002 |
| 7 | 168Er | FoxP3 | FJK-16s | eBioscience | 14-5773-82 |
| 8 | 165Ho | IFNγ | XMG1.2 | Bio X Cell | BE0055 |
| 9 | 154Sm | CD62L | MEL14 | Biolegend | 104402 |
| 10 | 157Gd | IL4 | 11B11 | Biolegend | 504102 |
| 11 | 159Tb | F4/80 | A3-1 | Bio-rad | MCA497G |
| 12 | 209Bi | CD11b | M1/70 | Homemade | / |
| 13 | 161Dy | iNOS | CXNFT | eBioscience | 14-5920-82 |
| 14 | 166Er | Arg1 | Polyclone | Fluidigm | 3166023B |
| 15 | 152Sm | CD11c | N418 | Biolegend | 117302 |
| 16 | 148Nd | Ly6c | HK1.4 | Biolegend | 128002 |
| 17 | 167Dy | CD49b | DX5 | Biolegend | 108902 |
| 18 | 147Sm | Ly6G | 1A8 | Biolegend | 127602 |
| 19 | 162Dy | TNFα | MP6-XT22 | Biolegend | 506302 |
| 20 | 163Dy | IL6 | MP5-20F3 | Biolegend | 504502 |
| 21 | 150Nd | IL10 | JES5-16E3 | Biolegend | 505002 |
| 22 | 155Gd | GM-CSF | MP1-31G6 | Biolegend | 505504 |
| 23 | 146Nd | CD38 | 90 | Biolegend | 102702 |
| 24 | 176Yb | IgM | RMM-1 | Biolegend | 406502 |
| 25 | 139La | Ki-67 | SolA15 | eBioscience | 14-5698-82 |
| 26 | 172Yb | PD-1 | 29F.1A12 | Biolegend | 135202 |
| 27 | 174Yb | CTLA-4 | UC10-4B9 | Biolegend | 106302 |
| 28 | 142Nd | MHC II | Y-3P | Bio X cell | BE0178 |
| 29 | 141Pr | CD24 | M1/69 | Biolegend | 101802 |
| 30 | 175Lu | Siglec-F | E50-2440 | BD Bioscience | 552125 |
| 31 | 169Tm | CD127 | A7R34 | Biolegend | 135002 |
| 32 | 89Y | CD45 | 30-F11 | Biolegend | 103102 |
